# Screening for Antifibrotic Compounds Using High Throughput System Based on Fluorescence Polarization

**DOI:** 10.3390/biology3020281

**Published:** 2014-04-10

**Authors:** Branko Stefanovic, Lela Stefanovic

**Affiliations:** College of Medicine, Florida State University, 1115 W. Call St., Tallahassee, FL 32306, USA; E-Mail: ela.stefanovic@med.fsu.edu

**Keywords:** fibrosis, antifibrotic drugs, RNA binding protein, fluorescence polarization

## Abstract

Fibroproliferative diseases are one of the leading causes of death worldwide. They are characterized by reactive fibrosis caused by uncontrolled synthesis of type I collagen. There is no cure for fibrosis and development of therapeutics that can inhibit collagen synthesis is urgently needed. Collagen α1(I) mRNA and α2(I) mRNA encode for type I collagen and they have a unique 5' stem-loop structure in their 5' untranslated regions (5'SL). Collagen 5'SL binds protein LARP6 with high affinity and specificity. The interaction between LARP6 and the 5'SL is critical for biosynthesis of type I collagen and development of fibrosis *in vivo*. Therefore, this interaction represents is an ideal target to develop antifibrotic drugs. A high throughput system to screen for chemical compounds that can dissociate LARP6 from 5'SL has been developed. It is based on fluorescence polarization and can be adapted to screen for inhibitors of other protein-RNA interactions. Screening of 50,000 chemical compounds yielded a lead compound that can inhibit type I collagen synthesis at nanomolar concentrations. The development, characteristics, and critical appraisal of this assay are presented.

## 1. Introduction

RNA binding proteins regulate key cellular processes, such as transcription, splicing, polyadenylation, translation, mRNA stability, subcellular partitioning of mRNA, formation of stress granules, and processing bodies [1,2,3,4,5,6]. These processes are often perturbed in diseases states and directly contribute to the pathogenesis [7]. However, there have been few attempts to target RNA binding proteins by drugs and high throughput screens to discover drugs that target RNA binding proteins are scarce [8,9]. The reason for this is that majority of RNA binding proteins bind multiple targets and very few of RNA binding proteins, such as histone stem-loop binding protein [10], iron responsive element binding protein [11], and LARP6 [12], have the specific target sequence, which they bind with high specificity. Another reason is the belief that the contact surfaces between an RNA binding protein and its cognate RNA are too large to be effectively disrupted by small molecules [13,14]. This article will describe the binding of LARP6 to the 5' stem-loop of type I collagen mRNAs, the biological consequences of this interaction and development of fluorescence polarization assay to screen for the inhibitory compounds. The initial characterization of one such compound will also be presented.

## 2. Binding of LARP6 to 5' Stem-Loop of Type I Collagen mRNAs

Type I collagen is the most abundant protein in human body. It is composed of three polypeptides, two α1(I) and one α2(I) polypeptides, which fold into rod-like heterotrimeric molecule. The heterotrimers of type I collagen are secreted into cellular medium, proteolytically cleaved, and polymerized into fibers [15]. The fibers are covalently cross linked to provide physical strength to the skin, bone, and tendons. However, in fibrosis there is excessive and unregulated synthesis of type I collagen in parenchymal organs, resulting in deposition of insoluble collagen fibrils throughout the organ, its insufficiency and death [16]. Fibrosis is almost always irreversible and progressive, however, there are no approved antifibrotic drugs and only supportive therapies are available. Biosynthesis of type I collagen is highly ordered process, because three polypeptides must be made in coordination and they must acquire proper posttranslational modification concomitant with folding. With the appearance of vertebrates a unique regulatory mechanism has evolved to support type I collagen synthesis. Collagen α1(I) and α2(I) mRNAs of all vertebrates have in their 5' UTR a unique stem-loop structure (5' stem-loop, 5'SL) [17,18,19]. A similar sequence is not found in any other mRNA, except collagen α1(III) mRNA, which encodes for type III collagen.

**Figure 1:**
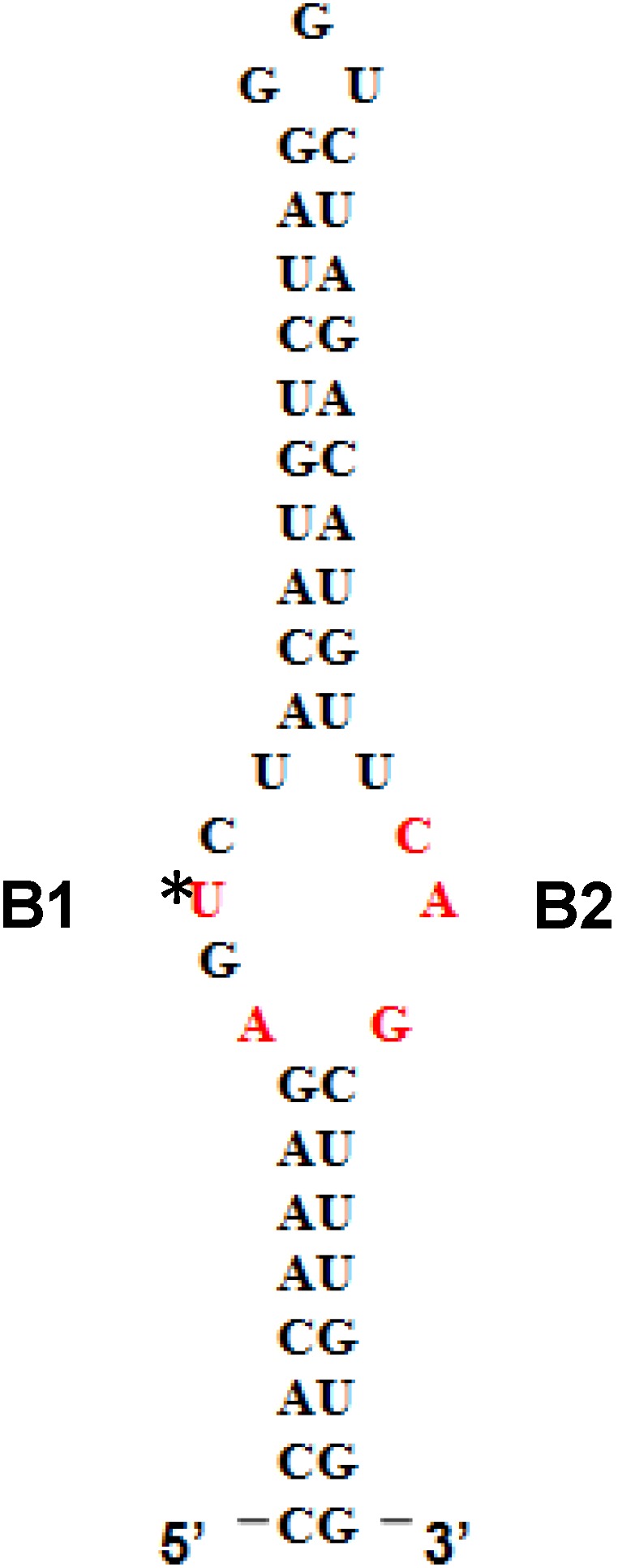
5' stem-loop of collagen mRNA. Sequence of human collagen α1(I) 5'SL. Nucleotides involved in binding LARP6 are indicated in red. For high throughput screening fluorescein label was added at the 5' end of this RNA. B1 and B2, single stranded regions.

5'SL consists of two double stranded stems, which flank a central single stranded bulge. 5'SL binds protein LARP6 with high affinity and specificity [12]. LARP6 does not bind any other RNA, making it the collagen mRNA specific RNA binding protein. For binding 5'SL, LARP6 needs the part of the protein termed the La-module, consisting of the La-domain and the adjacent RNA recognition motif (RRM). Binding of LARP6 is critical for high level of collagen synthesis, as seen in fibrosis, because mice carrying a mutation of the 5'SL in collagen α1(I) gene are resistant to development of hepatic fibrosis [20]. The binding affinity of LARP6 to 5'SL is in low nano-molar range [12]. LARP6 interacts with the single stranded regions of the 5'SL (B1 and B2 in Figure 1). While the La domain is a conserved motif shared by other members of the LARP superfamily, the RRM of LARP6 has very limited homology with the RRMs found in other RNA binding proteins [21]. The contacts between the 5'SL and LARP6 are mediated by the amino acids within the La domain and within the RRM, which are unique for LARP6 (our result). LARP6 serves as an adapter protein that shunts collagen mRNAs into the two pathways, one pathway leads to stabilization of collagen mRNA and the other pathway facilitate their translation. Binding of LARP6 to vimentin intermediate filaments prolongs the half life of collagen mRNAs [22]. The interaction of LARP6 with RNA helicase A [6], STRAP [23], and nonmuscle myosin filaments [24] increases the translatability of collagen mRNAs and couples translation of collagen α1(I) mRNA to that of α2(I) mRNA so that the corresponding peptides are produced in 2:1 ratio. Thus, by tethering accessory factors to collagen mRNAs, LARP6 plays a key role in regulating synthesis of type I collagen.

## 3. Binding of LARP6 as Target of Antifibrotic Drugs

In fibrosis collagen synthesis increases several hundred fold [25]. However, when knock in mice were created in which the 5'SL of collagen α1(I) mRNA was mutated, the animals were resistant to development of hepatic fibrosis and their liver cells responsible for the fibrotic changes were unable to upregulate type I collagen production [20]. These mice developed normally, suggesting that the 5'SL/LARP6 mechanism is primarily responsible for collagen synthesis in fibrosis, but not for constitutive collagen synthesis. Based on the facts that: (1) 5'SL is the specific element of collagen mRNA; (2) LARP6 binds only collagen 5'SL; and (3) the binding is critical for collagen synthesis in reactive fibrosis, the interaction between LARP6 and 5'SL is an ideal target for development of antifibrotic drugs. Most current attempts to discover antifibrotic drugs are focused on attenuating inflammation under laying fibrosis [26,27] or targeting TGFβ signaling pathway [28,29,30]. The approach described here is the first attempt to discover a drug directly and specifically targeting collagen biosynthesis.

## 4. RNA Binding Proteins and High Throughput Screening

**Figure 2:**
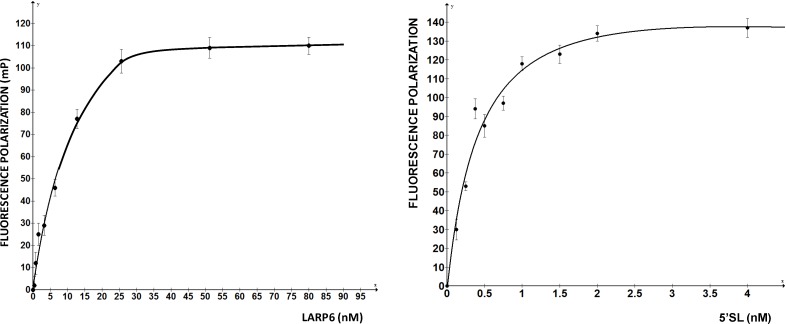
**Left** panel: saturation of 1 nM of fl-5'SL RNA with increasing concentrations of LARP6. **Right** panel: saturation of 25.6 nM LARP6 with increasing concentrations of fl-5'SL RNA. FP of free fl-5'SL RNA was subtracted from the total FP to show only the protein dependent FP.

Fluorescence polarization (FP) is a method that measures the size of a fluorophore [31,32]. The higher the size of the fluorophore the greater the FP, thus, formation and dissociation of an RNA-protein complex can be monitored if one component is fluorescently labeled. As fluorescently labeled RNA can be easily obtained and FP measurement is easily automated, the method is ideal for high throughput monitoring of RNA-protein interactions. The critical component of the assay is the protein and, practically, only recombinant protein expressed in *E. coli* can give sufficient quantities for high throughput assays. However, two aspects must be kept in mind when using bacterially expressed mammalian RNA binding proteins. First, does the bacterially expressed protein make the same contacts with the RNA as the natural protein? It is likely that in a pure two-component system the bacterially expressed protein will bind its target, but does it make exactly the same contacts as the native protein. If not, then using such preparation for screening of drugs that will interfere with the binding of native protein is meaningless. One way to test the specificity is to make single point mutations in the RNA and analyze them for binding. If the recombinant protein binds the mutants with the same relative specificity as the native protein, it can be assumed that it is folded correctly, as well as that posttranslational modifications do not contribute to the specificity. This is reasonable approach for strictly sequence specific RNA binding proteins, like LARP6, but may present a problem for proteins that bind degenerate sequences or homopolymers. Second, what is the fraction of active protein present in the preparation? It is not uncommon that up to 99% of the protein may be biologically inactive [33,34,35]. One way to estimate this is to first titrate a fixed amount of RNA with increasing concentrations of protein and then titrate a fixed amount of protein with increasing concentrations of RNA. The assumption is that RNA is folded properly and will select only the active conformations of the protein. Figure 2 shows such titrations for LARP6 binding to 5'SL. While titration of the fixed amount of 5'SL RNA (1 nM) with increasing amounts of LARP6 gave a Kd of 7 nM, the titration of 25.6 nM of LARP6 with increasing concentrations of 5'SL RNA gave a Kd of 0.33 nM. This translates that only about 5% of LARP6 molecules in the preparation are in active conformation.

For the experiments in Figure 2, His-tagged LARP6 containing only the La-module was purified by a single step using Ni-NTA agarose resin. Attempts to increase the purity by additional chromatographic steps resulted in an increase in the fraction of inactive molecules. Therefore, at least for LARP6, the number of purification steps should be kept at minimum, because small amounts of impurities may be less detrimental than presence of >95% of inactive molecules. For the FP assay it is important to estimate the fraction of active molecules, as large amounts of inactive protein tend to form aggregates, what may interfere with the FP readings. This may be especially problematic for low affinity RNA binding proteins, where larger quantities of protein are needed. We have obtained satisfactory results in high throughput screening with the LARP6 preparations similar to the one shown in Figure 2. The titrations will also determine the concentration of protein that saturates a given amount of RNA, without either component being in excess. This condition is optimal for screenings based on FP. Based on these considerations, the next chapter will describe the application of the assay for screening of chemical compounds that inhibit binding of LARP6 to 5'SL RNA.

## 5. FP as High Throughput Assay for Binding of LARP6 to 5'SL RNA

**Figure 3:**
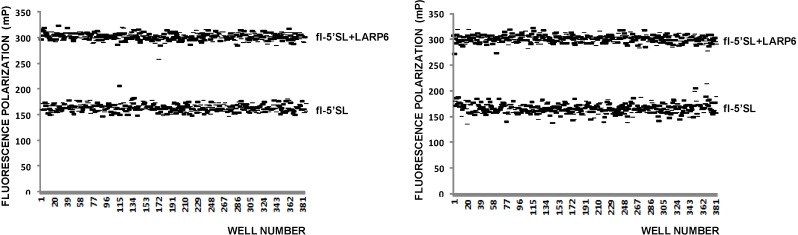
FP of free fl-5'SL RNA and fl-5'SL RNA bound to LARP6. Measurements were done in 384-well plates immediately after filling the wells (**left** panel) and 6 h later (**right** panel) and individual readings are plotted. The Z-value was calculated from the left panel according to standard formula [38].

Since FP is proportional to the size of the fluorophore [31,36,37], fluorescently labeled but unbound RNA will also give a FP reading that is lower than that of the RNA-protein complex. 5'SL RNA is 53 nt long and when labeled by fluorescein at the 5' end 1 nM solution has FP of 150–160 mPU. Addition of saturating amounts of LARP6 increases the FP to 280–320 mPU (Figure 3). These two values represent the upper and lower limits of the assay (Figure 3, top panel) and can used to calculate the Z-value of the assay using the formula Z = 3(σ_p_ + σ_n_)/µ_p_ − µ_n_[38], where σ_p_ and σ_n_ are standard deviations of FP of fully bound 5'SL RNA and free 5'SL RNA and µ_p_ and µ_n_ are the mean values, respectively. By using FP readings obtained from a 384-well plate (Figure 3, left panel) a Z value of 0.5 was calculated, indicating that FP is an acceptable high throughput assay for binding of LARP6.

**Figure 4:**
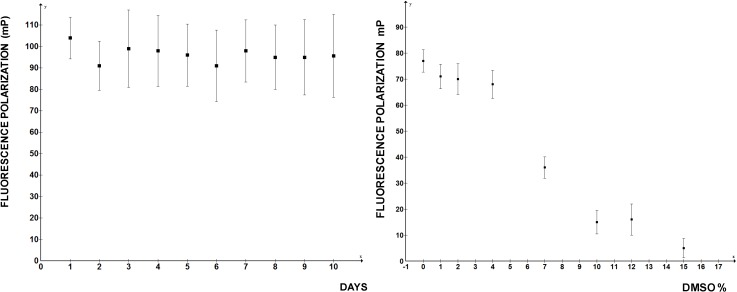
Day-to-day reproducibility of the FP assay and tolerance to DMSO concentrations. **Left** panel: independent readings were performed on 10 consecutive days. **Right** panel: increasing concentrations of DMSO were added to the reaction.

After incubation of 6 h at RT the FP readings of the same plate were repeated; they showed more variability between the wells after the prolonged incubation (Figure 3, right panel), but there was no massive dissociation of the 5'SL/LARP6 complex, indicating that the assay is stable. Day-to-day variability of FP readings was 10%–15% and the presence of DMSO in reaction up to 4% did not significantly diminish the FP readings (Figure 4). At higher DMSO concentrations FP rapidly deteriorated, so if the compounds to be screened are dissolved in DMSO, this limits the amount that can be added into the reaction. Thus, by the important standards of high throughput assays FP measurements of LARP6 binding to 5'SL RNA qualifies as a good assay. There is no reason to believe that a similar assay could not be developed for any other sequence specific RNA binding protein.

## 6. Application of the Assay

The assay was developed to screen a library of chemical compounds for a compound that can displace LARP6 from fl-5'SL RNA, as detected by a decrease in FP. To show that dissociation of LARP6 can be detected, competition experiments were performed in which unlabeled 5'SL RNA was added in increasing concentrations into the reaction containing fl-5'SL RNA and LARP6. The decrease in FP in concentration dependent manner indicated dissociation of LARP6 from fl-5'SL RNA and redistribution onto the competitor 5'SL RNA, while unrelated RNA showed no effect. We were able to compete out 80% of the bound LARP6 with 250-fold molar excess of the competitor. This verified that the assay can detect dissociation of LARP6 and that it is suitable for high throughput screening based on the inhibition of binding.

Based on the titration curves (Figure 2), for the screening of chemical library we premixed 1 nM fl-RNA and 25.6 nM LARP6. Total volume of 25 µL was distributed per well of 384 well plates and 50,000 drug like compounds were added at final concentration of 40 µM. This compound library was purchased from ChemBridge, INC., San Diego, CA, USA and all compounds in this library conformed to the Lipinski rules. The final DMSO concentration in the reaction was 2%. FP was measured in duplicates using Biotek Synergy H1 plate reader and the mean and standard deviation of FP for each plate was calculated. The values that were lower by 2 standard deviations from the mean were considered positive hits. There were on average 1–2 positive hits per 384-well plate, but such large number indicated that many hits were false positive hits. The main reason for false positive hits was that many compounds possess intrinsic fluorescence. This is a general problem with fluorescent assays [31,37]. The spectral overlap increased the total fluorescence intensity in many wells. FP, as read in our assay, was not independent on fluorescence intensity, but it decreased proportionally to the total intensity, resulting in false positive hits. To identify the compounds that increase total fluorescence intensity it is mandatory to read the total fluorescence intensity together with the FP.

**Figure 5:**
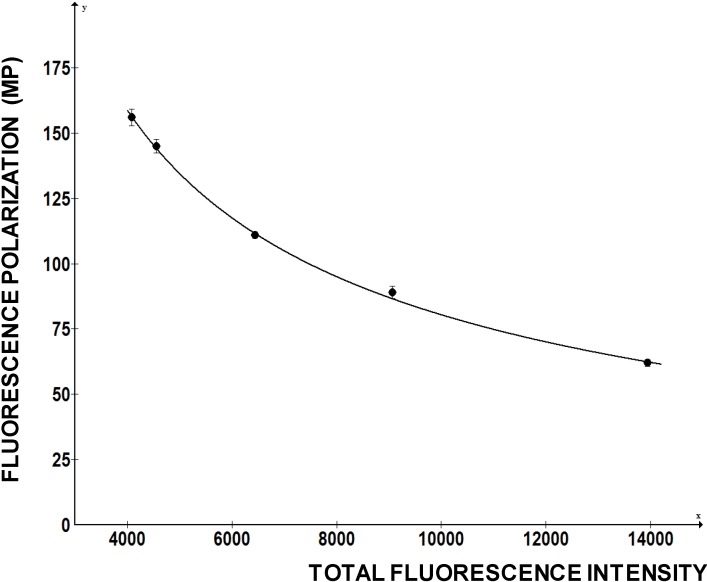
Change in FP as function of fluorescence intensity. Increasing amounts of free fluorescein was added to the fl-5'SL RNA and FP was plotted as function of total fluorescence intensity.

To investigate how FP of a fluorescein labeled RNA change with the total fluorescence intensity we added increasing amounts of free fluorescein to the fixed amounts of fl-5'SL RNA and measured FP (Figure 5). Fluorescein is a small molecule with molecular weight of 376 D, so it only increased the total fluorescence intensity without contributing to the FP. However, FP readings of the fl-5'SL RNA decreased proportionally to the total fluorescence intensity and could be reduced by >50% just by adding the dye (Figure 5).

**Figure 6:**
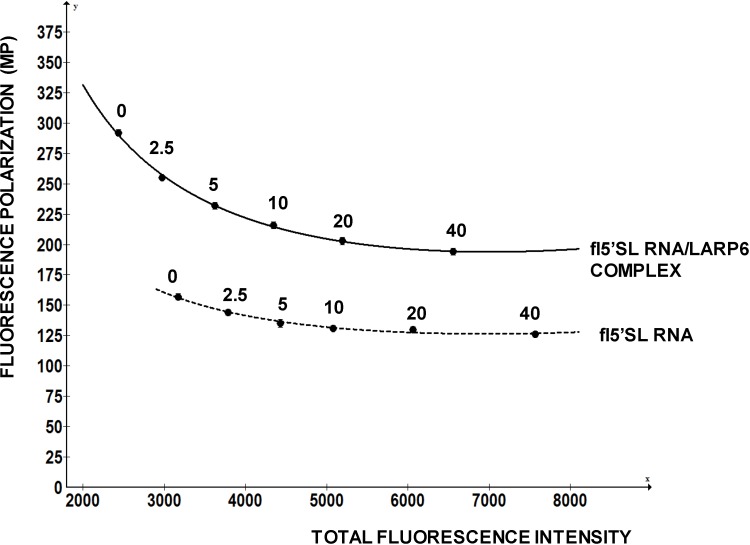
Re-evaluation of a positive hit. A compound that has intrinsic fluorescence was added in the different concentrations (nM, indicated above the curves) to 1 nM of free fl-5'SL RNA (dotted line) or to 1 nM fl-5'SL RNA in complex with LARP6 (full line) and FP was plotted as function of total fluorescence intensity.

This example clearly indicates that a chemical compound with intrinsic fluorescence can negatively affect FP readings and present itself as a positive hit. This is a major drawback of the assay and, as high concentrations of compounds are used in the initial screens, the measurements based on decrease in FP must be carefully re-evaluated. Figure 6 shows a re-evaluation of one compound that has intrinsic fluorescence, but that is also active in displacing LARP6 form 5'SL. This compound has excitation at 370 nM and maximal emission at 420 nM, which is distant from the excitation and emission of fl-5'SL RNA (480/520). It was added in concentrations from 0 to 40 nM to either 1 nM of free fl-5'SL RNA or to 1 nM fl-5'SL RNA bound to LARP6 and FP was plotted as function of total fluorescence intensity. Although, the compound increased total fluorescence intensity several fold, it marginally decreased FP of the fl-5'SL RNA alone (dotted line). However, it significantly reduced the FP of fl-5'SL/LARP6 complex, suggesting the effectiveness in dissociating the complex. We recommend similar evaluation of the positive hits that are associated with a significant increase in total fluorescence intensity.

We re-screened the positive hits obtained in our initial screen by the strategy similar as above. The positive hit compounds were added at concentrations from 40 µM to 1 µM to fl-5'SL RNA alone or to LARP6/fl-5'SL RNA complex and FP was measured. Compounds that were able to selectively decrease the FP of LARP6/fl-5'SL RNA complex were considered true hits. By this re-evaluation procedure we ended up with 19 compounds (out of 50,000 compounds screened, 0.04%) that were active in dissociating LARP6 from fl-5'SL RNA. These compounds were directly tested in a cell-based assay for the ability to inhibit of type I collagen synthesis.

## 7. Validation of the Hits in Cell Based Assay

**Figure 7:**
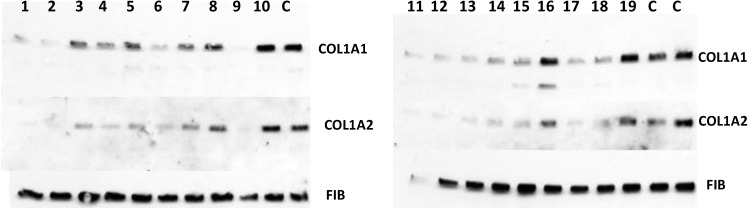
Antifibrotic activity of the compounds identified in FP in cell based assay. The compounds were added to human fibroblasts at 10 µM and collagen α1(I) (COL1A1) and α2(I) (COL1A2) polypeptides were analyzed in cellular medium by Western blot. Loading control, fibronectin (FIB).

Collagen secreted into the extracellular medium is responsible for fibrosis, so measuring the ability of cells to secrete type I collagen is a pertinent way to predict the antifibrotic potential of a drug [39,40]. Therefore, the compounds identified in the screen were added at 10 µM to the culture of human lung fibroblasts [12] and secretion of collagen α1(I) and α2(I) polypeptides into the cellular medium was analyzed by Western blot. Figure 7 shows that 15 of the 19 positive hits from the screen were moderately effective in inhibiting collagen production by fibroblasts (compounds **1**–**8**, **11**–**15**, **17**, **18**) and that three were completely ineffective (compounds **10**, **16**, **19**), as compared to controls (DMSO). Secretion of fibronectin was not affected, suggesting an effect specific for type I collagen. However, one compound (compound **9**) showed dramatic inhibition at 10 µM concentration and was designated as a lead compound. It had similar structure to the three other compounds (compounds **2**, **6**, and **13**) that were less potent, but clearly active.

Thus, the screening was successful in identifying compounds that are inhibitory to collagen production in a cell-based assay. The lead compound could be classified to the same structural group with three other compounds, suggesting that the method has selected for compounds of similar structures. The molecular weight of the active compounds ranged from 250 to 400, the octanol/water partition coefficient (clogP) ranged from 0.58 to 4.72, the total polar surface area (t-PSA) ranged from 42 to 94.7 and the solubility, expressed as logSw, was between −2.11 and −5.54. The lead compound was further tested at lower concentrations in other types of fibroblasts and was effective in 500–250 nM range.

**Figure 8:**
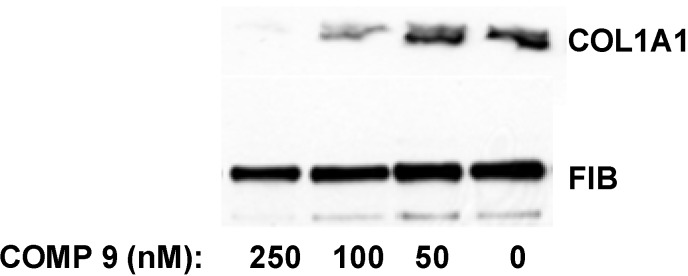
Inhibition of collagen expression in HSCs. Compound **9** was added to HSCs at day two in culture and incubation continued for five additional days. The medium with the compound was changed daily. Collagen α1(I) polypeptide (COL1A1) was measured in the cellular medium at day seven by Western blot. Loading control, fibronectin (FIB).

Hepatic stellate cells (HSCs) are liver cells responsible for excessive collagen production in liver fibrosis [41,42]. They are present in normal liver in quiescent state, but when isolated and subjected to culture *in vitro*, they spontaneously activate within seven days and increase their collagen synthesis 50–100 fold [43,44,45]. We isolated HSCs from normal rat livers and cultured them *in vitro*. After two days in culture the cells were still quiescent and expressed type I collagen at barely detectable levels. The transformation into the activated state was initiated at day three to four in culture and the terminal activation into myofibroblast-like cells was achieved after seven to eight days. At this time point collagen expression was maximal and 50–100 fold higher than at day two. When the lead compound was added to quiescent HSCs in culture at day two after isolation and the cells were subjected to activation in the presence of the compound for additional five days, the collagen synthesis was inhibited by 50% at 100 nM and almost completely abolished at 250 nM (Figure 8). Thus, the compound was even more potent in preventing type I collagen production by HSCs during their activation *in vitro*.

## 8. Mechanism of Action of the Lead Compound

**Figure 9:**
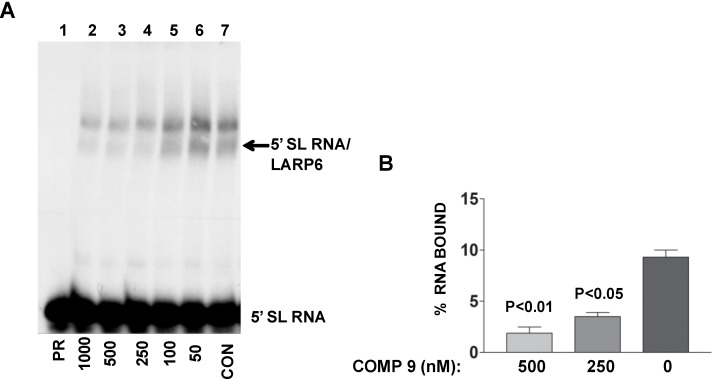
Binding of LARP6 in cells treated with the lead compound. (**A**) The compound was added to lung fibroblasts at the nM concentrations indicated and cell extracts were used in gel mobility shift assay with radiolabeled 5'SL as probe. PR, 5'SL RNA probe alone, CON, untreated cells. Migration of free RNA and 5'SL/LARP6 complexes is indicated. (**B**) The amount of LARP6/RNA complexes formed was quantified and expressed as percentage of the input RNA. The results are from two independent experiments. Error bars, ±1 SD.

The lead compound was identified by its ability to decrease FP of fl-5'SL RNA/LARP6 complex. However, is its activity in the cells also due to dissociation of LARP6 from the 5'SL of collagen mRNAs? To verify this we treated lung fibroblasts with various concentrations of the compound, prepared cell extracts and analyzed the binding of endogenous LARP6 to radiolabeled 5'SL RNA as probe using gel mobility shift assay. As shown in Figure 9A, the ability of endogenous LARP6 to interact with 5'SL RNA was diminished in concentration dependent manner (lanes 2–6), as compared to the untreated cells (lane 7). By quantifying the LARP6/RNA complexes, we estimated that the inhibition was 60%–70% (Figure 9B). This clearly demonstrated that the compound can inhibit binding of LARP6 to 5'SL RNA when added to the cells. Whether the lead compound interacts with LARP6 or with 5'SL RNA or both remains to be determined. However, this example clearly demonstrates that RNA-protein interactions in the cell can be inhibited by small molecules.

## 9. Conclusions

A FP assay that was used to screen for chemical compounds that can dissociate LARP6 from a complex with collagen 5'SL RNA is described. This interaction is critical for high level of collagen synthesis and the inhibitors may be potential antifibrotic drugs. During development of the assay several observations were made that may have general implications for development of similar assays for other RNA binding proteins. (1) RNA binding proteins purified from *E. coli* are mostly inactive in specifically binding the RNA, only small fraction of the molecules recognize the RNA in the sequence specific manner; (2) This may not an impediment for high throughput screening if active molecules are present in sufficient amounts to give a good FP signal; (3) Excessive purification may further decrease the fraction of active molecules, so it is a trade off that has to be considered on individual basis; (4) Intrinsic fluorescence of the chemical compounds that are screened may decrease FP readings, without affecting the RNA/protein complex; (5) The effect takes place even if the fluorescence spectrum of the compound is only partially overlapping with the spectrum of the fluorophore carried by the RNA; (6) This is the main cause of false positive hits. To recognize these artifacts fluorescence intensity readings must be performed together with the FP readings. The compounds with such properties must be additionally tested. (7) Identification of structurally related compounds in the screen is a good indicator that the screen has yielded functional molecules.

Based on our experience, it is possible to use FP to identify small molecules that can dissociate RNA binding proteins from the RNA. Most of the compounds identified in our screen were effective in inhibiting collagen production, suggesting that such screens may be highly productive. In conclusion, this work has established FP as a legitimate tool for high throughput screening of compounds affecting RNA binding properties of proteins.
